# Bacterial Diversity Profiling of the New Zealand Parasitic Blowfly Lucilia sericata Based on 16S rRNA Gene Amplicon Sequencing

**DOI:** 10.1128/MRA.00257-21

**Published:** 2021-05-13

**Authors:** Nikola Palevich, Paul H. Maclean, Luis Carvalho, Ruy Jauregui

**Affiliations:** a AgResearch Limited, Grasslands Research Centre, Palmerston North, New Zealand; Indiana University, Bloomington

## Abstract

Here, we present a 16S rRNA gene amplicon sequence data set and profiles demonstrating the bacterial diversity of larval and adult Lucilia sericata, collected from Ashhurst, New Zealand (May 2020). The two dominant genera among adult male and female *L. sericata* were *Serratia* and *Morganella* (phylum *Proteobacteria*), while the larvae were also dominated by the genera *Lactobacillus*, *Carnobacterium*, and *Lactococcus* (phylum *Firmicutes*).

## ANNOUNCEMENT

Members of Calliphoridae (blowflies) are economically important for medical and veterinary management worldwide (1). Larvae of this fly invade their animal host, feed on tissues and excretions, and progressively cause severe skin disease, commonly referred to as flystrike (myiasis) (2, 3). Currently, control relies heavily on the prophylactic application of long-acting chemicals to all sheep, but this approach is increasingly under threat due to the development of resistance to current treatments. Lucilia sericata NZ_LucSer_NP (4) was selected for microbiome assessment as a representative of a New Zealand field strain of *L. sericata*. In this study, we investigated the bacterial microbiomes of *L. sericata* larvae, adult males, and adult females to gain a better understanding of the microbial communities and especially symbionts to blowflies that could lead to entirely novel treatments against flystrike and blowfly control.

The *L. sericata* specimen larvae were collected from a farm site in the Ashhurst area in New Zealand (40°18′S, 175°45′E). Species identification and rearing of the blowflies on beef liver as a protein source and a 10% sugar solution were done according to Dear (5). Lab-reared separate pools of larval, adult male, and adult female *L. sericata* blowflies were washed twice in sterile phosphate-buffered saline (PBS; pH 7.4) to remove surface-adherent bacteria, snap-frozen in liquid nitrogen, and transferred to –80°C storage prior to DNA extraction. High-molecular-weight genomic DNA was isolated from *L. sericata* pooled samples of 100 larvae as well as 10 entire adult males and females per replicate (*n* = 5 for each). Genomic DNA was prepared for metagenomic 16S rRNA gene amplicon sequencing of the V3-V4 hypervariable region using a modified phenol-chloroform protocol recently described for complex samples, such as parasitic roundworms (6, 7), fastidious anaerobic rumen bacteria (8–10), and spore-forming psychrotolerant *Clostridium* sp. isolated from spoiled meat (11, 12). A DNA library was prepared using the 16S V3-V4 rRNA library preparation method (Illumina, Inc., San Diego, CA) according to the manufacturer’s instructions (13) and sequenced on the Illumina MiSeq platform with the 2 × 250-bp paired-end (PE) reagent kit v2, producing a total of 5,208,027 PE raw reads.

The processing of the amplicon reads followed a modified version of the pipeline described in reference 14. The reads produced by the sequencing instrument were paired using the program FLASH2 v2.2.00 (15). Paired reads were then quality trimmed using Trimmomatic v0.38 (16). The trimmed reads were reformatted as fasta, and the read headers were modified to include the sample name. All reads were compiled into a single file, and Mothur v1.45.2 (17) was used to remove reads with homopolymers longer than 10 nucleotides (nt) and to collapse the reads into unique representatives. The collapsed reads were clustered using Swarm v2 (18). The clustered reads were filtered based on their abundance, keeping representatives that were (i) present in one sample with a relative abundance of >0.1%, (ii) present in >2% of the samples with a relative abundance of >0.01%, or (iii) present in 5% of the samples at any abundance level. The selected representatives were annotated using QIIME 2 v2017.4 (19) with the SILVA database v138 (20). The annotated tables were then used for downstream statistical analysis. Sample and sequence data are summarized in Table 1.

**TABLE 1:** Details of all *Lucilia sericata* samples used in this study and information for sequencing reads

Sample^*a*^	Life cycle stage	No. of raw reads	No. of quality-filtered reads	SRA accession no.
Adult_Male_1	Adult male	437,598	437,584	SRR13779722
Adult_Male_2	Adult male	391,709	391,686	SRR13779721
Adult_Male_3	Adult male	372,187	372,165	SRR13779720
Adult_Male_4	Adult male	342,606	342,588	SRR13779719
Adult_Male_5	Adult male	442,342	442,324	SRR13779718
Adult_Female_1	Adult female	389,860	389,841	SRR13779716
Adult_Female_2	Adult female	376,696	376,677	SRR13779715
Adult_Female_3	Adult female	422,959	422,938	SRR13779714
Adult_Female_4	Adult female	359,511	359,497	SRR13779713
Adult_Female_5	Adult female	298,290	298,284	SRR13779712
L3_Larvae_1	Larvae L3	326,910	326,904	SRR13779711
L3_Larvae_2	Larvae L3	270,953	270,946	SRR13779710
L3_Larvae_3	Larvae L3	290,495	290,492	SRR13779709
L3_Larvae_4	Larvae L3	276,269	276,265	SRR13779708
L3_Larvae_5	Larvae L3	209,642	209,638	SRR13779707

a All samples were collected in March 2020 from the Ashhurst area in New Zealand (40°18′S, 175°45′E).

**FIG 1:**
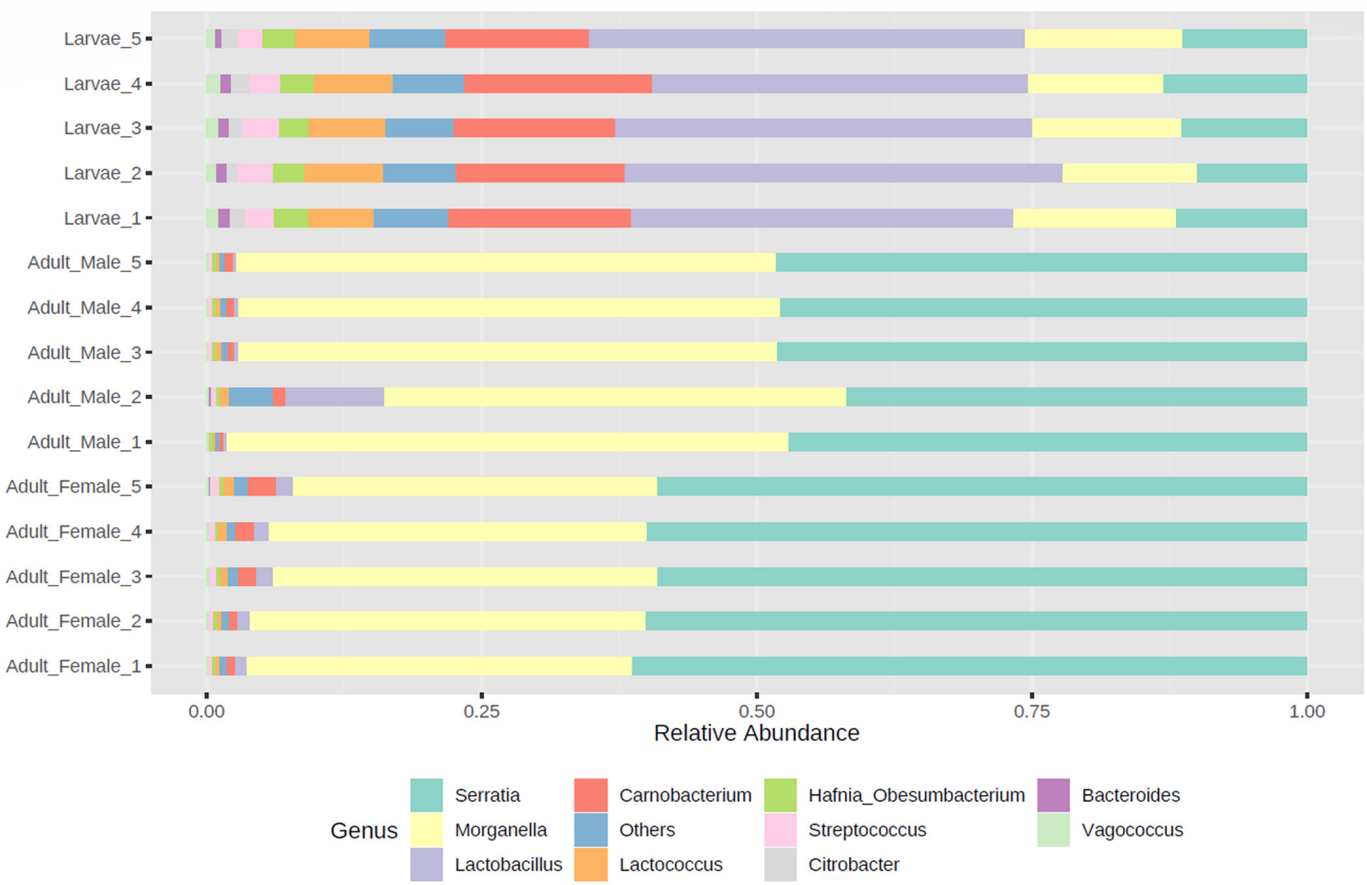
The taxonomic composition of the dominant bacteria of New Zealand *L. sericata*. Relative abundance of the dominant bacterial genera obtained from 16S rRNA sequencing of *L. sericata* field strain NZ_LucSer_NP larval, adult male, and adult female samples. Genera with a relative abundance of less than 1% and unassigned amplicon sequence variants were grouped together as others.

In all samples, the predominant phylum was *Proteobacteria* (Fig. 1) and the predominant genera were *Serratia* and *Morganella*, while the larvae were also dominated by *Lactobacillus*, *Carnobacterium*, and *Lactococcus* (phylum *Firmicutes*). The metagenomic 16S rRNA gene amplicon sequencing of *L. sericata* field strain NZ_LucSer_NP reported here is a valuable resource for future studies investigating the role of bacteria in flystrike. In order to improve the phylogenetic resolution of the microbial community structures and improve our knowledge of flystrike caused by *L. sericata*, future efforts should focus on the generation of amplicon sequencing data from numerous locations around New Zealand and across a wider range of blowfly species (21).

### Data availability.

The 16S rRNA gene amplicon sequence data have been deposited in the GenBank Sequence Read Archive (SRA) under the BioProject accession number PRJNA667961.
